# Effects of Smartphone-Based Remote Interventions on Dietary Intake, Physical Activity, Weight Control, and Related Health Benefits Among the Older Population With Overweight and Obesity in China: Randomized Controlled Trial

**DOI:** 10.2196/41926

**Published:** 2023-04-28

**Authors:** Na Zhang, Mingzhu Zhou, Muxia Li, Guansheng Ma

**Affiliations:** 1 Department of Nutrition and Food Hygiene, School of Public Health, Peking University Beijing China

**Keywords:** overweight and obesity, remote interventions, older population, dietary intake, physical activity, weight management, health, mobile phone

## Abstract

**Background:**

Traditional health management requires many human and material resources and cannot meet the growing needs. Remote medical technology provides an opportunity for health management; however, the research on it is insufficient.

**Objective:**

The objective of this study was to assess the effects of remote interventions on weight management.

**Methods:**

In this randomized controlled study, 750 participants were randomly assigned to a remote dietary and physical activity intervention group (group DPI), remote physical activity intervention group (group PI), or control group (group C). At baseline (time 1), day 45 (time 2), and day 90 (time 3), data were collected, including data on dietary intake, physical activity, indexes related to weight control, and health benefits.

**Results:**

A total of 85.6% (642/750) of participants completed the follow-up. Compared with group C, group DPI showed a significant decrease in energy intake (−581 vs −82 kcal; *P*<.05), protein intake (−17 vs −3 g; *P*<.05), fat intake (−8 vs 3 g; *P*<.05), and carbohydrate intake (−106.5 vs −4.7 g; *P*<.05) at time 3. Compared with time 1, groups DPI and PI showed a significant decrease in cereal and potato intake (*P*<.05). Compared with time 1, the physical activity levels related to transportation (group PI: 693 vs 597 metabolic equivalent [MET]–min/week, group C: 693 vs 594 MET-min/week; *P*<.05) and housework and gardening (group PI: 11 vs 0 MET-min/week, group C: 11 vs 4 MET-min/week; *P*<.05) in groups PI and C were improved at time 3. Compared with groups PI and C, group DPI showed a significant decrease in weight (−1.56 vs −0.86 kg and −1.56 vs −0.66 kg, respectively; *P*<.05) and BMI (−0.61 vs −0.33 kg/m^2^ and −0.61 vs −0.27 kg/m^2^, respectively; *P*<.05) at time 2. Compared with groups PI and C, group DPI showed a significant decrease in body weight (−4.11 vs −1.01 kg and −4.11 vs −0.83 kg, respectively; *P*<.05) and BMI (−1.61 vs −0.40 kg/m^2^ and −1.61 vs −0.33 kg/m^2^, respectively; *P*<.05) at time 3. Compared with group C, group DPI showed a significant decrease in triglyceride (−0.06 vs 0.32 mmol/L; *P*<.05) at time 2. Compared with groups PI and C, group DPI showed a significant decrease in systolic blood pressure (−8.15 vs −3.04 mmHg and −8.15 vs −3.80 mmHg, respectively; *P*<.05), triglyceride (−0.48 vs 0.11 mmol/L and −0.48 vs 0.18 mmol/L, respectively; *P*<.05), and fasting blood glucose (−0.77 vs 0.43 mmol/L and −0.77 vs 0.14 mmol/L, respectively; *P*<.05). There were significant differences in high-density lipoprotein cholesterol (−0.00 vs −0.07 mmol/L; *P*<.05) and hemoglobin A_1c_ (−0.19% vs −0.07%; *P*<.05) between groups DPI and C.

**Conclusions:**

Remote dietary and physical activity interventions can improve dietary intake among participants with overweight and obesity, are beneficial for weight control, and have potential health benefits.

**Trial Registration:**

Chinese Clinical Trial Registry ChiCTR1900023355; https://www.chictr.org.cn/showproj.html?proj=38976

## Introduction

### Background

The aging trend of the world’s population is increasingly prominent. In 2015, the absolute number of people aged ≥60 years worldwide was 900 million; this is expected to increase to 1.4 billion by 2030 and to 2.1 billion by 2050, and it may exceed 3.2 billion by 2100 [1]. In the past 30 years, because of changes in lifestyle, environment, and other factors, the rate of overweight and obesity has increased rapidly worldwide and become one of the biggest public health challenges in the 21st century [2]. In 2016, among adults aged >18 years, 13% were obese (BMI≥30 kg/m^2^) and 39% were overweight (25 kg/m^2^≤BMI<30 kg/m^2^), and the absolute number of people with overweight and obesity exceeded 1.9 billion [3]. The rate of overweight and obesity in the older population is also rapidly rising. According to the data from The Chinese Health and Retirement Longitudinal Study, the rate of overweight (24 kg/m^2^≤BMI<28 kg/m^2^) among the older population in China increased from 26.14% in 2011 to 30.14% in 2015, and the rate of obesity (BMI≥28 kg/m^2^) increased from 9.58% to 10.75% [4]. Studies have shown that overweight or obesity is an independent risk factor for chronic diseases such as type 2 diabetes, hypertension, and cardiovascular diseases [5]. In 2002, when the adult overweight and obesity rate reached 29.9%, the direct economic cost was US $3.1 billion [6]. It has been estimated that the direct economic burden of adult chronic diseases caused by overweight and obesity will increase to US $7.2 billion by 2030. Among the risk factors for overweight, obesity, and chronic diseases, most factors, with the exception of genetic factors, can be changed by adopting healthy dietary behaviors and lifestyles to prevent or delay the occurrence and development of chronic diseases [7]. Even in the older population, appropriate physical activity and reasonable diet can produce great health benefits. Therefore, it is imperative to implement weight and health management, actively control risk factors, abandon unhealthy lifestyles, practice healthy lifestyles, and enhance self-management awareness.

Traditional weight and health management methods mainly include health education and clinical management, which require many human and material resources and are relatively inefficient and expensive. The popularization of traditional health management is also limited by medical resources, time, and space, and traditional health management cannot meet the needs of populations with chronic diseases or obesity. Therefore, it is necessary to explore long-term, simple, widespread, economic, and effective weight and health management interventions. In April 2021, We Are Social and Hootsuite jointly released the Digital 2021 April Global Statshot Report [8], which stated that by April 2021, there were 5.27 billion mobile phone users, 4.72 billion internet users, and 4.33 billion social media users, with internet users accounting for >60% of the world’s total population. The emerging remote and mobile medicine technology has broken the limitations of time and space in traditional health management and medical treatment [9], and the adoption of remote and mobile medicine in health management has become a trend. Smartphones are convenient to carry and are an indispensable tool for communication. Therefore, remote health management using smartphones has become an important way to administer remote and mobile medicine with great potential. As a carrier of remote and mobile health technology, smartphones are installed with third-party apps and used in weight and health management. They have also become an important technical medium of eHealth development in recent years and have been gradually accepted by the public [10].

In the remote and mobile health management system, mobile terminals such as smartphones and smart devices (eg, activity trackers, sphygmomanometers, and blood glucose meters) can be connected via wireless (eg, Bluetooth) or wired (eg, USB) networks to collect and transmit data on physiological information, such as users’ heart rate, walking steps, dietary intake, body temperature, blood pressure, blood glucose, and body weight, to smartphone apps [11-14]. Users can also input the measurement data into the app by themselves, track health information remotely, and receive intervention measures to improve their weight and health outcomes. At present, remote management apps for weight and health management mainly include health monitoring and assessment, medication management, health information dissemination, health education, risk factor intervention, guidance, feedback, and follow-up. In the study conducted by Thomas et al [15], participants with overweight and obesity aged 19 to 70 years with a BMI of 25 to 50 kg/m^2^ used the *DailyBurn* smartphone app to record their daily dietary intake, physical activity, and weight, and feedback on energy intake and expenditure was provided to the participants automatically every day. In the 12th week of the intervention, the dietary intake of the participants decreased, and the average weight loss was 8.4 kg. In the 24th week of the intervention, the average weight loss was 10.9 kg, indicating that the use of a remote management system to intervene in the risk factors of chronic diseases was helpful in forming a healthy lifestyle [15]. A randomized controlled trial conducted in Finland recruited patients with diabetes whose hemoglobin A_1c_ (HbA_1c_) was >6.5% and *randomly divided* them into an intervention group and a control group (group C). The patients in the intervention group were provided with mobile phones installed with the Monica app and were required to measure blood glucose in the morning 3 days a week, measure body weight 1 day a week, and upload data on health monitoring to the Monica app. Then, the Monica app automatically analyzed the data and provided feedback to the user in the form of graphics and text. After 10 months of intervention, the results indicated that HbA_1c_ decreased by 0.4% (95% CI −0.67% to −0.14%) and body weight decreased by 2.1 kg (95% CI −3.6 to −0.6) in the intervention group [16]. Another researcher randomly divided older patients with type 2 diabetes living in a community in Shanghai into an intervention group and group C. Both groups were provided routine chronic disease management support and daily basic treatment. On this basis, the intervention group were also provided the smartphone app “Diabetes Care APP 2.0” for management, including function modules of blood glucose detection, health education, and physician-patient interaction. The study found that the intervention group’s self-management ability and glucose metabolism indicators improved [17]. A randomized controlled intervention study explored the effect of mobile phone apps including function modules of disease science popularization, medication reminders, diet guidance, rehabilitation training, and other functions on older patients with type 2 diabetes. The results showed that the levels of fasting blood glucose (FBG), 2-hour postprandial blood glucose, and glycosylated hemoglobin in the intervention group were significantly lower than those in group C (*P*<.05); the scores on the diabetes self-management behavior scale (Summary of Diabetes Self-Care Activities) in the observation group were significantly higher than those in group C at 6 and 12 months after discharge (*P*<.05); and the incidence of complications in the intervention group was significantly lower than that in group C (*P*<.05) [18]. A study conducted by Laranjo et al [19] found that interventions using a combination of mobile apps and physical activity trackers could effectively promote physical activity. A systematic review of 25 studies showed that for middle-aged and older people with a BMI of ≥25 kg/m^2^, a short-term (6-month) weight management plan combined with wearable devices may be more effective than regular weight management [20]. Logan et al [21] conducted a randomized controlled trial in middle-aged and older patients with diabetes and hypertension and found that 51% of the patients in the intervention group who used mobile apps with both blood pressure monitoring equipment and function modules of information feedback reduced their blood pressure to the target value recommended by the guidelines (<130/80 mm Hg), whereas only 31% of the patients in group C reached the standard. In a study, middle-aged and older patients with hypertension under standardized management by the Hypertension Research Institute of the Second Affiliated Hospital of Baotou Medical College used intelligent sphygmomanometers to monitor their blood pressure and smartphone apps to monitor and manage their blood pressure. After 12 months, the systolic blood pressure (SBP) and diastolic blood pressure (DBP) of the patients significantly decreased (*P*<.001) [22]. Sun et al [23] used the remote “Internet plus” internet-based management mode to intervene in older patients with hypertension aged ≥60 years for 6 months. The results of the follow-up study showed that compared with the ordinary blood pressure monitoring mode, the remote “Internet plus” interactive management mode helped improve the blood pressure status and drug compliance of the patients [23].

It is necessary to conduct a relevant study to evaluate the impacts of a remote intervention and remote management system for the weight and health management of older people. Better health management for older people is fundamental to saving medical and socioeconomic resources and promoting the happiness and quality of life of older people and their families. With the increase in the aging population, the prevalence of chronic diseases among older people in China is increasing, and the prevention and control of chronic diseases are facing serious challenges. In recent years, although the investment in health services has grown, China’s medical and health service institutions and health personnel for older people remain limited, and there is room for improvement in the social security and medical system for the prevention and control of chronic diseases among the older population. The ability to provide health services to the older population remains insufficient. With the popularity of remote and intelligent devices and the rapid development of remote mobile internet technology, it is possible to use remote management systems to provide dynamic health monitoring, health management, and other services for older people. To fill the gap in the field of remote health management for older people, it is necessary and important to conduct relevant studies to assess the impact of remote health management and intervention, explore the remote health management mode, and provide a scientific basis for remote health management so as to improve the quality of life of the older population, control the development of chronic diseases, reduce the burden on society and families, and promote healthy aging.

### This Study

A remote management system mode that included “health information collection—health assessment—guidance and feedback—follow-up” was used in this study. Nutritional professionals, physical activity instructors, and participants were instructed on the use of the remote management system to provide real-time monitoring of health data, a scientific health management plan, feedback, guidance, follow-up, and social support for the participants. In this 3-month randomized controlled trial, the effects of a remote dietary and physical activity intervention on dietary intake, physical activity, weight control, and related health benefits were explored. The feasibility and effectiveness of the remote management system mode for managing the older participants’ weight and health were evaluated to provide a scientific basis for establishing the mode and measures of remote health management among the older population.

## Methods

### Study Design and Hypothesis

This was a randomized controlled trial. The hypothesis for this study is that the remote management system mode is feasible for the older population and helpful in improving dietary intake and physical activity and further promoting weight loss and the improvement of health effect indicators.

### Sample Size Calculation

Based on the formula *N=2(Z_α⁄2_+ Z_β_)^2^ σ^2^/δ^2^*, to achieve a power (1-β) of .9 with an α of .05, a *σ* of 0.9, and a *δ* of 0.3, 189 participants were required. In the formula, *σ* (0.9) and *δ* (0.3) were based on a study related to the effects of a mobile medical technology on the weight management of adults with overweight and obesity [24].

Considering the loss to follow-up rate of 20%, 237 participants were needed in each group (189 / 0.8 = 237). In this study, 3 groups were formed for comparison. Thus, the total sample size was at least 711 participants (237 *×* 3 = 711).

### Participants

Participants were recruited through community posters and social media information releases on the internet and WeChat (Tencent Holdings Ltd). The inclusion criteria were as follows: between 60 and 80 years of age, BMI≥24 kg/m^2^, not having participated in any activity or study related to weight control and management, having a smartphone, and being able to use a smartphone. The exclusion criteria were as follows: being diagnosed with cognitive dysfunction, schizophrenia, depression, or other mental disorders; having a pacemaker or other medical electronic device in the body; having difficulty walking; having a history of obesity surgery; having a history of alcoholism; and not willing to change lifestyle. In the study, a total of 1119 participants were recruited. Of them, 67.02% (750/1119) participants met the inclusion criteria.

### Ethics Approval

The study protocol and instruments were reviewed and approved by the Ethical Review Committee of the Peking University in 2018. The identification code is IRB00001052-18039. The study was conducted in accordance with the guidelines of the Declaration of Helsinki. Before the start of the study, all the participants read and signed an informed consent form voluntarily. The study protocol was registered in the Chinese Clinical Trial Registry (ChiCTR190023355).

### Randomization

Using the random number table method, 750 participants were randomly divided into 3 groups: a remote dietary and physical activity intervention group (group DPI), remote physical activity intervention group (group PI), and group C, with 250 (33.3%) participants in each group.

### Remote Management System

**Figure 1:**
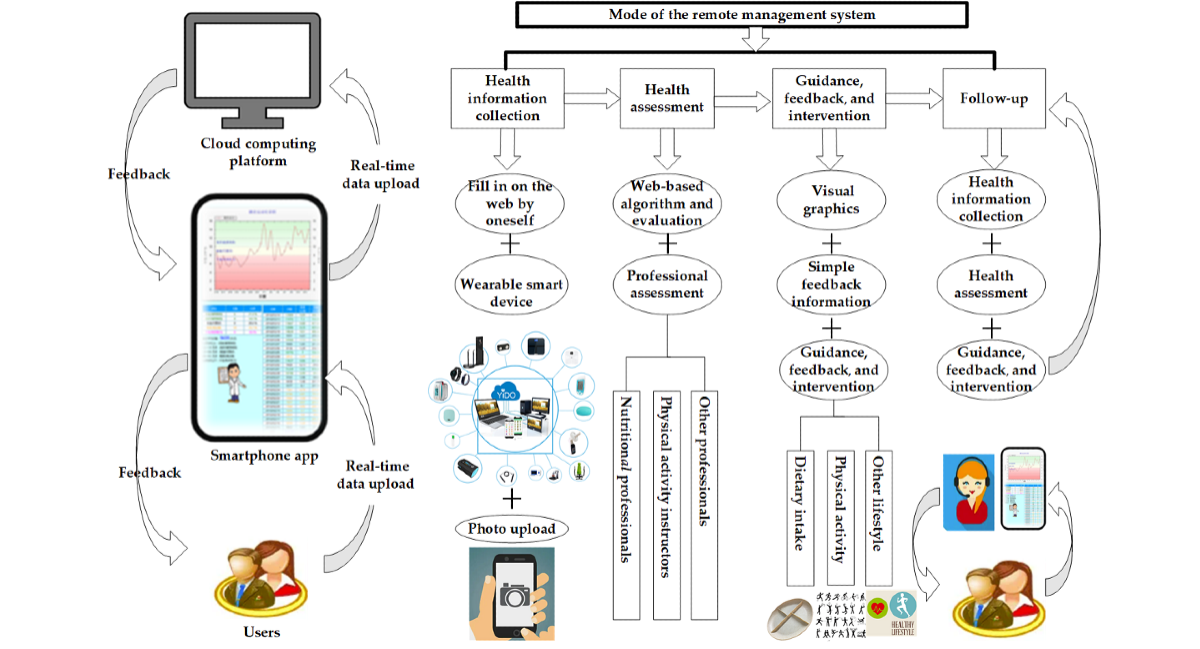
Remote management system used in the study.

A remote management system smartphone app (YIDO Health app, YIDO Artificial Intelligence Technology [Shandong] Co, Ltd) was used in this study (Figure 1). The remote management system in the study was mainly divided into 4 steps: “health information collection—health assessment—guidance and feedback—follow-up.” The smartphone-based remote management system remotely connected the system users with nutritional professionals or physical activity instructors. Nutritional professionals or physical activity instructors could obtain the relevant user information through the cloud, including basic personal health information, dietary intake, physical activity, weight, waist circumference, hip circumference, and blood pressure. System users could set their own health goals (such as target weight) after logging into the app and connect smart devices such as weighing scales and activity trackers to the smartphone through Bluetooth to automatically upload health monitoring data to the app. Users wore an activity tracker and regularly measured their weight, waist circumference, hip circumference, and blood pressure. After comparing the data with the target, the app generated visual graphics (bar chart or broken line chart) and simple feedback information, such as “poor (Further efforts are needed),” “ordinary (You can be better),” and “good (You should continue to keep it up).” Users could also upload their daily food logs and pictures of standardized meals and physical activity information through the app. Nutritional professionals or physical activity instructors could offer assessments, personalized communication, and guidance through the WeChat social group or phone based on the relevant information collected by the app. At the same time, users’ personalized dietary and lifestyle behaviors could be improved by following the guidance provided by the app. In addition, if users had any questions, they could consult their nutritional professionals or physical activity instructors through the app at any time.

### Interventions

#### Overview

The specific intervention measures in the study included a remote dietary intervention and remote physical activity intervention based on the remote management system on the smartphone app. Please refer to Table 1 for further details.

**Table 1:** Details of the contents of the intervention measures.

Interventions and contents of the intervention measures				Implementer
**Group DPI^a^**				
	**Health record establishment**			
		Fill in the health information form on the web	Participants	
	**Remote dietary intervention**			
		Evaluate the daily dietary intake of the participants	Nutritional professionals	
		Upload the food logs and photos of standardized dietary plate to the app every day	Participants	
		Low-energy diet recipes	Participants	
		Web-based health education about the importance of weight management and benefits of reasonable dietary intake	Nutritional professionals	
		Provide one-on-one personalized dietary guidance and feedback to the participants through the WeChat social group or phone according to their age, gender, weight, food intake, chronic disease situation, choice of food type, and portion size 3 to 5 times a week	Nutritional professionals	
		Establish a WeChat management social group including the participants; the group owner reminds and urges the participants to upload and record daily dietary intake information	Nutritional professionals	
	**Remote physical activity** **intervention**			
		Evaluate the physical activity of the participants	Physical activity instructors	
		Wear an activity tracker connected to the smartphone app through Bluetooth and upload information about walking steps every day. Information about other types of physical activity should be filled in on the web and uploaded to the app by the participants.	Participants	
		Web-based health education about the importance of weight management and benefits of regular active physical activity	Physical activity instructors	
		Encourage and advise the participants to complete at least 20 minutes of resistance physical activity (such as squatting, push-ups, and weight lifting) or 20 minutes of aerobic physical activity (such as walking, jogging, and dancing) or walk 6000 steps every day. Then, provide the participants with one-on-one personalized dietary guidance and suggestions through the WeChat social group or phone according to their age, gender, weight, food intake, chronic disease situation, and choice of physical activity type and advice on the intensity and duration of physical activity 3 to 5 times a week	Physical activity instructors	
		Establish a WeChat management social group including the participants; the group owner reminds and urges the participants to upload and record daily physical activity information	Physical activity instructors	
	**Weight monitoring**			
		Record the body weight in the fasting state every day and upload the data to the app	Participants	
		Measure the waistline and hipline every week and upload the data to the app	Participants	
	**Blood pressure monitoring**			
		Measure the blood pressure every week and upload the data to the app	Participants	
	**Blood glucose monitoring**			
		Measure the fingertip blood glucose every week and upload the data to the app	Participants	
	**Medication reminder**			
		Collect the medication information of those with abnormal blood pressure and blood glucose and regularly remind them of their medication	Nutritional professionals	
**Group PI^b^**				
	**Health record establishment**			
		Fill in the health information form on the web	Participants	
	**Remote physical activity** **intervention**			
		Evaluate the physical activity of the participants	Physical activity instructors	
		Wear an activity tracker connected to the smartphone app through Bluetooth and upload information about walking steps every day; information about other types of physical activity should be filled in on the web and uploaded to the app by the participants	Participants	
		Web-based health education about the importance of weight management and benefits of regular active physical activity	Physical activity instructors	
		Encourage and advise the participants to complete at least 20 minutes of resistance physical activity (such as squatting, push-ups, and weight lifting) or 20 minutes of aerobic physical activity (such as walking, jogging, or dancing) or walk 6000 steps every day. Then, provide the participants with one-on-one personalized dietary guidance and suggestions through the WeChat social group or phone according to their age, gender, weight, food intake, chronic disease situation, and choice of physical activity type and advice on the intensity and duration of physical activity 3 to 5 times a week	Physical activity instructors	
		Establish a WeChat management social group including the participants; the group owner reminds and urges the participants to upload and record daily physical activity information	Physical activity instructors	
	**Weight monitoring**			
		Record the body weight in the fasting state every day and upload the data to the app	Participants	
		Measure the waistline and hipline every week and upload the data to the app	Participants	
	**Blood pressure monitoring**			
		Measure the blood pressure every week and upload the data to the app	Participants	
	**Blood glucose monitoring**			
		Measure the fingertip blood glucose every week and upload the data to the app	Participants	
	**Medication reminder**			
		Collect the medication information of those with abnormal blood pressure and blood glucose and regularly remind them of their medication	Nutritional professionals	
**Group C^c^**				
		Distribute a health education book on a reasonable diet to the participants	Researchers	

^a^Group DPI: remote dietary and physical activity intervention group.

^b^Group PI: remote physical activity intervention group.

^c^Group C: control group.

#### Remote Dietary Intervention

At baseline (time 1), web-based health education was offered. The risks of overweight and obesity, importance of weight management, and benefits of reasonable dietary intake were explained to the participants. The participants were recommended to prepare kitchen scales, salt control spoons, and oil control spoons or pots. During the study period, the participants were asked to upload their food logs and photos with standardized dietary plate to the app every day to track and record their food intake. The standardized meal was divided into 4 parts: cereals and potatoes (25%); fruits (25%); vegetables (35%); and livestock, poultry, and aquatic products (15%), as shown in Figure 1. Low-energy diet recipes were provided to the study participants. In addition, the nutritional professionals provided one-on-one personalized dietary guidance and feedback to the participants through the WeChat social group or phone according to their age, gender, weight, food intake, chronic disease situation, choice of food type, and portion size 3 to 5 times a week according to the compliance of the participants.

#### Remote Physical Activity Intervention

At time 1, web-based health education was offered. The risks of overweight and obesity, importance of weight management, and benefits of regular physical activity were explained to the participants. During the study period, the participants were encouraged and advised to complete at least 20 minutes of resistance physical activity (eg, squatting, push-ups, and weight lifting) or 20 minutes of aerobic physical activity (eg, walking, jogging, and dancing) or walk 6000 steps every day. After completing the daily physical activity, related photos and the types and time of physical activity were uploaded to the app by participants. In addition, the physical activity instructors provided the participants with one-on-one personalized dietary guidance and suggestions through the WeChat social group or phone according to their age, gender, weight, food intake, chronic disease situation, and choice of physical activity type and advice on the intensity and duration of physical activity 3 to 5 times a week according to the compliance of the participants. However, considering the typical physiological functions of older participants, it was not mandatory for them to complete all physical activities. The activity tracker was required to track and record their number of walking steps.

The participants in group DPI received both remote dietary and physical activity interventions for 3 months. Group PI received a remote physical activity intervention for 3 months. In addition, during the study period, the participants in groups DPI and PI were required to monitor and measure their weight, waist circumference, hip circumference, blood pressure, and blood glucose every week and upload the data to the app. The participants in group C did not receive an intervention and could maintain their previous lifestyle, but each was given a health education book on a reasonable diet.

### Study Procedure

**Figure 2:**
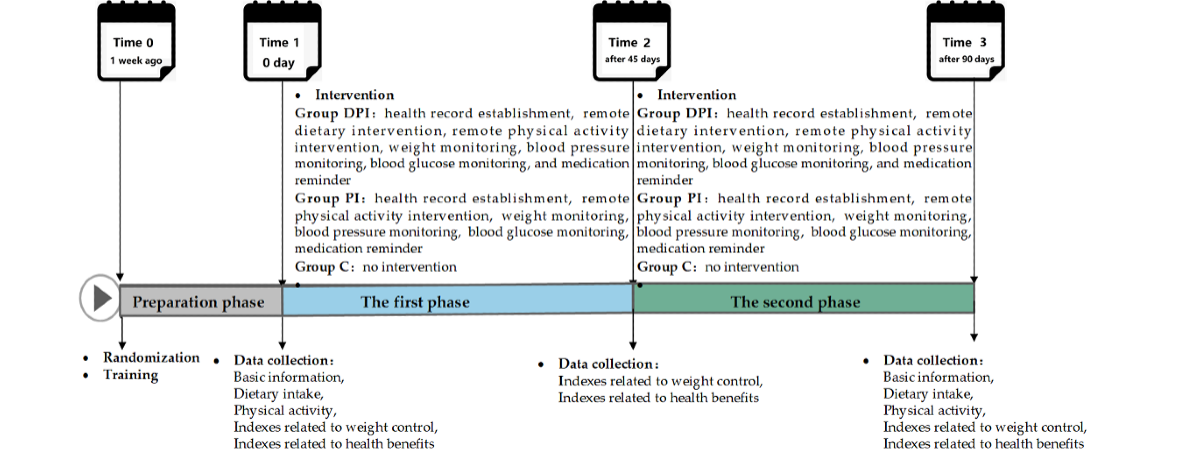
The study procedure. Group C: control group; Group DPI: remote dietary and physical activity intervention group; Group PI: remote physical activity intervention group.

The intervention period of the study was 3 months, including the preparation phase (from time 0 to time 1), the first phase (from time 1 to time 2), and the second phase (from time 2 to time 3). The study procedure is illustrated in Figure 2.

#### The Preparation Phase

After recruiting and screening the participants based on the inclusion and exclusion criteria, the uniformly trained researchers introduced the purpose, procedures, benefits, potential risks, inconveniences, and discomfort of the study to the participants. The participants signed an informed consent form when they agreed to participate. The participants were randomly divided into 3 groups: group DPI, group PI, and group C. Next, the participants in groups DPI and PI were trained on the smartphone health management app, activity tracker, and other intervention measurements that needed to be coordinated. After opening an app account number for the participants, the researchers helped them download and log into the app; taught them to use the app, activity tracker, and standardized meal; and instructed them on the methods and steps of monitoring their weight, blood pressure, blood glucose, etc. They allowed the participants to practice using the app to record and upload their data and photos on dietary intake and physical activity, as well as their weight, blood pressure, blood glucose, and other monitoring data. Group C participants maintained their previous lifestyle.

#### The First Phase

On the morning of time 1, the participants were asked to arrive at a designated place in the fasting state at 7 AM. Venous blood samples were collected from the elbow, and body weight, waistline, and hipline were measured. At the same time, the researchers instructed them to complete the food frequency questionnaire and physical activity questionnaire one-on-one. From time 1 to time 2 (day 45), the participants in group DPI received remote dietary and physical activity intervention, and the participants in group PI received remote physical activity intervention. The weight, waistline, hipline, blood pressure, and blood glucose of the participants in both groups were monitored. The participants in group C did not receive any intervention, and each of them was given a health education book on a reasonable diet at time 1.

#### The Second Phase

At time 2, the participants were asked to arrive at the designated place in the fasting state at 7 AM. Venous blood samples were collected from the elbow, and body weight, waistline, and hipline were measured. From time 2 to time 3 (day 90), the participants in group DPI received a remote dietary and physical activity intervention, and the participants in group PI received a remote physical activity intervention. The weight, waistline, hipline, blood pressure, and blood glucose of the participants in both groups were monitored. The participants in group C did not receive any intervention. Finally, at time 3, the participants were asked to arrive at the designated place in the fasting state at 7 AM. Venous blood samples were collected from the elbow, and body weight, waistline, and hipline were measured. At the same time, the researchers instructed them to complete the food frequency questionnaire and physical activity questionnaire one-on-one.

### Dietary Intake

Food intake was recorded and estimated using a semiquantitative food frequency questionnaire. The type, frequency, and amount of food intake were recorded. The amount of food intake was estimated by the participants by referring to food models and photographic models [25]. Nutrient intake from food was estimated by the researchers using the Chinese Food Composition Table [26].

### Physical Activity

A physical activity questionnaire was used to record the physical activity of the participants. The questionnaire was developed based on the International Physical Activity Questionnaire (shortened version) [27] and revised in accordance with expert comments. The questionnaire consisted of 27 items, including items about the type, frequency, and time of various types of physical activities. The participants were classified based on whether their physical activity was of low intensity, middle intensity, or high intensity according to the criteria in the study by Fan et al [28]. If any 1 of the following 2 criteria was met, the level of physical activity was classified as high: (1) within a week, the number of days of high-intensity physical activity were ≥3, and the total amount of weekly physical activity was ≥1500 metabolic equivalent [MET]–min/week and (2) within a week, the number of days of physical activity, including physical activities of high-, moderate-, and low-intensity levels, was ≥7, and the total amount of weekly physical activity was ≥3000 MET-min/week. Meeting any 1 of the following 3 criteria classified as a moderate physical activity level: (1) performing all kinds of high-intensity physical activities for at least 20 minutes every day, and the number of days of high-intensity physical activity was ≥3; (2) performing all kinds of moderate intensity or walking activities for at least 30 minutes every day, and the number of days of moderate intensity or walking activity was ≥5; and (3) within a week, the number of days of physical activity, including physical activities of high-, moderate-, and low-intensity levels, was ≥5, and the total amount of weekly physical activity was ≥600 MET-min/week. If any 1 of the following 2 criteria was met, the level of physical activity was classified as low: (1) no activity was reported and (2) some activities were reported, but they did not meet the aforementioned criteria for moderate and high physical activity levels.

### Indexes Related to Weight Control

Weight and height were measured by trained investigators according to relevant standards and procedures. Participants were to wear light clothing and no footwear, participants’ height was measured twice with 0.1 cm accuracy, and weight was measured twice with 0.1 kg accuracy using a height-weight meter (HDM-300, Huaju). BMI (kg/m^2^) was measured as weight (kg)/height squared (m^2^). Waistline and hipline were measured according to relevant standards and procedures by trained investigators using a MyoTape measurer (Accu Measure). Waistline and hipline were measured twice to the nearest 0.1 cm.

### Indexes Related to Health Benefits

Blood pressure (SBP and DBP) was measured twice by the researchers to the nearest 2 mm Hg using a desktop mercury sphygmomanometer (Yuwell Danyang). The 2 measurements were taken at 15-minute intervals. Approximately 5 mL of fasting cubital venous blood was collected from the participants. HbA_1c_ was determined using a glycosylated hemoglobin analyzer (HLC-723G7, TOSOH) with supporting reagents (glycosylated hemoglobin assay kit, Wako Industry Co, Ltd). Fasting plasma glucose level was determined using a biochemical analyzer (Cobas C 501, Roche) with supporting reagents (blood glucose measurement kit, Jiancheng Bioengineering Research Institute). total cholesterol (TC), triglyceride (TG), high-density lipoprotein cholesterol (HDL-C), and low-density lipoprotein cholesterol (LDL-C) were determined using the enzyme colorimetry method with supporting reagents (reagents kit, Jiancheng Bioengineering Research Institute).

### Blinding

Throughout the study, the participants were identified by a code number, and the data collectors, result evaluators, and data analysts were unaware of the grouping of the study participants.

### Quality Control

As for the unification of study procedure and training, standardized training was provided to the researchers to familiarize them with all the procedures. Unified training was conducted to inform the participants of how the app should be used, how the corresponding questionnaires should be filled in, how relevant data should be uploaded, and the procedures they needed to follow. To avoid contamination between the different groups, remote dietary and physical interventions were conducted one-on-one through WeChat or telephone communication, rather than through the mobile phone app used in the study. In addition, the study participants could not register with the app by themselves. Registration had to be done by the background personnel, and an account number had to be provided before logging in. Therefore, the participants in groups DPI and PI could use the app, whereas those in group C could not. Throughout the trial, all the participants were prohibited from using health management apps or technologies other than the app in this study. After the study began, the entire process was strictly supervised and controlled by special quality-control researchers.

### Statistical Analysis

SAS (version 9.2; SAS Institute Inc) was used. Qualitative data are presented as n (%), and data were compared using a chi-square test. Continuous quantitative data conforming to normal distribution are presented as mean (SD), and values were compared using the method of repeated measurement analysis (ANOVA). Continuous quantitative data not conforming to normal distribution are presented as median (IQR), and values were compared at time 1 and time 3 using the Mann-Whitney *U* test and compared among the 3 groups using the Kruskal-Wallis test. Two-sided *P*<.05 was considered as statistically significant.

## Results

### Follow-up of the Participants

**Figure 3:**
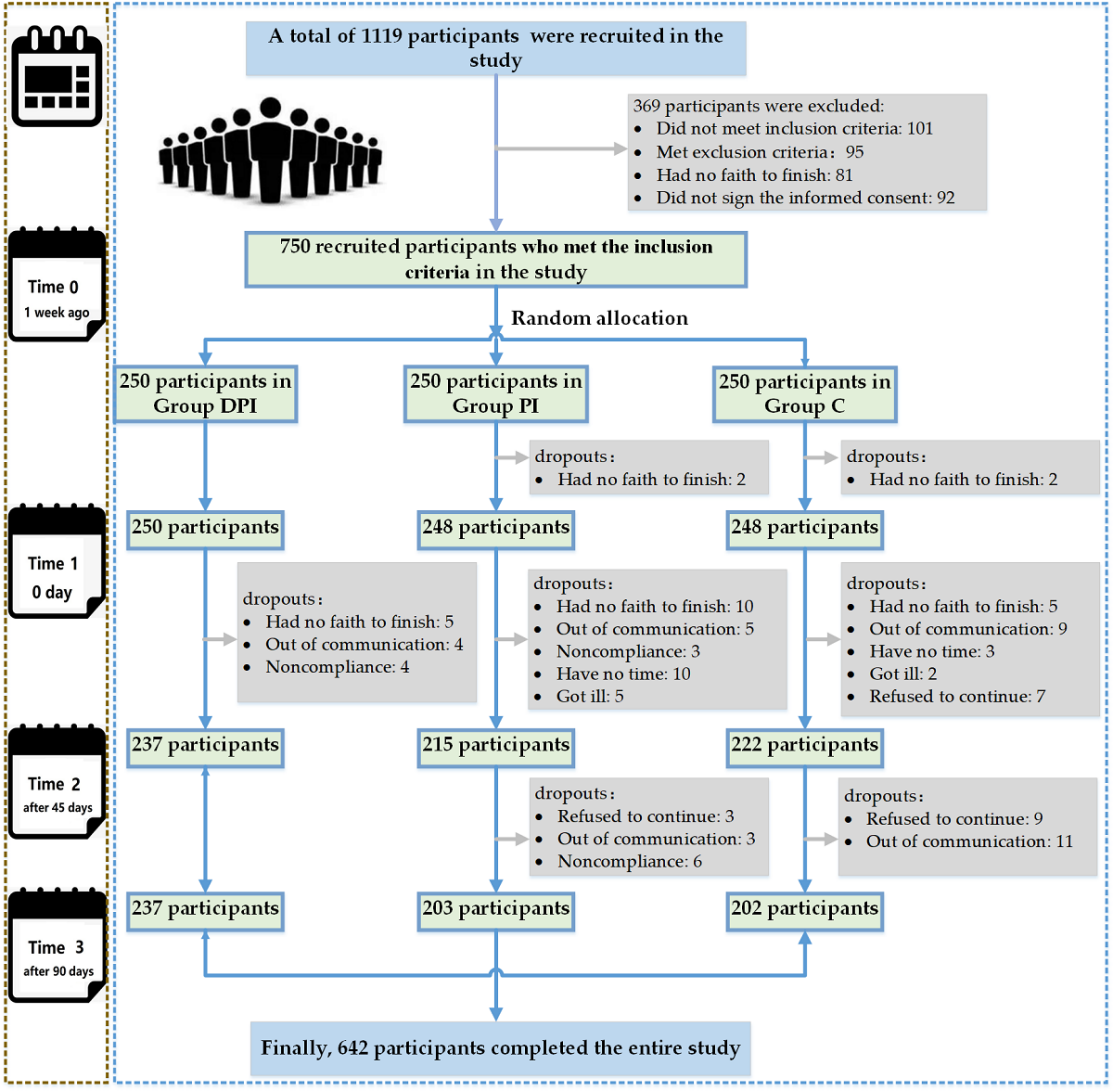
Follow-up flowchart of the participants from the beginning to the end of the study.

Among the recruited 750 participants who met the inclusion criteria, 746 (99.5%) participants completed the baseline test at time 1, including 100% (250/250) in group DPI, 99% (248/250) in group PI, and 99% (248/250) in group C. At time 2, a total of 89.9% (674/750) of participants completed the follow-up test, including 94.8% (237/250) in group DPI, 86% (215/250) in group PI, and 88.8% (222/250) in group C. At time 3, a total of 85.6% (642/750) of participants completed the follow-up, including 94.8% (237/250) in group DPI, 81.2% (203/250) in group PI, and 80.8% (202/250) in group C. Details are illustrated in Figure 3.

### Characteristics of the Participants

Among the 746 participants at time 1, the mean age was 70.1 (SD 5.3) years, 344 (46.1%) were female participants, and 402 (53.9%) were male participants. Age, height, weight, BMI, waistline, waist-hip ratio, distribution of educational level, and smoking habit showed no discrepancy among the participants in group DPI, group PI, and group C (table 2). The characteristics of the participants are listed in Table 2.

**Table 2:** Characteristics of the participants.

Characteristics			Total (n=746)		Group DPI^a^ (n=250)		Group PI^b^ (n=248)		Group C^c^ (n=248)		*F* test (*df*) or chi-square (*df*)			*P* value	
**Sex^d^, n (%)**												0.0 (745)^d^			.98
	Male	344 (46.1)		114 (45.6)		115 (46.4)		115 (46.4)							
	Female	402 (53.9)		136 (54.4)		133 (53.6)		133 (53.6)							
Age (year)^e^, mean (SD)			70.1 (5.3)		70.3 (5.5)		69.7 (5.3)		70.2 (5.3)		0.856 (745)^e^			.43	
Height (cm)^e^, mean (SD)			159.61 (7.95)		159.69 (7.97)		159.67 (8.13)		159.45 (7.79)		0.067 (745)^e^			.94	
Weight (kg)^e^, mean (SD)			70.62 (9.57)		70.55 (9.08)		70.47 (9.67)		70.85 (10.02)		0.111 (745)^e^			.90	
BMI (kg/m^2^)^e^, mean (SD)			27.67 (2.63)		27.61 (2.26)		27.60 (2.86)		27.80 (2.72)		0.455 (745)^e^			.63	
Waistline (cm)^e^, mean (SD)			94.78 (8.00)		93.93 (8.14)		95.17 (7.95)		95.23 (7.89)		2.094 (745)^e^			.12	
Waist-hip ratio^e^, mean (SD)			0.93 (0.06)		0.92 (0.06)		0.93 (0.06)		0.93 (0.06)		2.155 (745)^e^			.12	
**Marital status^d^, n (%)**												30.2 (745)^d^			<.001^f^
	Living alone	123 (16.5)		34 (13.6)		47 (19)		42 (16.9)							
	Married	623 (83.5)		216 (86.4)		201 (81)		206 (83.1)							
**Education level^d^, n (%)**												0.4 (745)^d^			.99
	Elementary school or below	140 (18.8)		46 (18.4)		46 (18.5)		48 (19.4)							
	Middle or high school	456 (61.1)		153 (61.2)		150 (60.5)		153 (61.7)							
	College degree or above	150 (20.1)		51 (20.4)		52 (21)		47 (19)							
**Smoking^d^, n (%)**												7.4 (745)^d^			.11
	Smoker	111 (14.9)		28 (11.2)		40 (16.1)		43 (17.3)							
	Former smoker	105 (14.1)		35 (14)		42 (16.9)		28 (11.3)							
	Nonsmoker	530 (71)		187 (74.8)		166 (66.9)		177 (71.4)							

^a^Group DPI: remote dietary and physical activity intervention group.

^b^Group PI: remote physical activity intervention group.

^c^Group C: control group.

^d^Values were compared using chi-square test.

^e^Values were compared using the method of repeated measurement analysis (ANOVA).

^f^Statistically significant differences: *P*<.05.

### Changes in Dietary Intake

At time 3, the daily total energy intake (*P*<.001), protein intake (*P*<.001), fat intake (*P*=.01), and carbohydrate intake (*P*<.001) of the participants in group DPI decreased. The daily fat intake of the participants in group PI decreased (*P*=.048), and the total energy intake (*P*=.12), protein intake (*P*=.07), and carbohydrate intake (*P*=.09) showed no statistical difference. There was no statistical difference in the total energy intake (*P*=.79) and intake of 3 major energy-supplying nutrients in group C (protein: *P*=.99; fat: *P*=.16; and carbohydrate: *P*=.36).

At time 3, the changes in the total energy intake (−581 vs −82 kcal; *P*<.05), protein intake (−17 vs −3 g; *P*<.05), fat intake (−8 vs 3 g; *P*<.05), and carbohydrate intake (−107 vs −5 g; *P*<.05) of the participants in group DPI were statistically different compared with the participants in group C. The changes in the total energy intake (−581 vs −125 kcal; *P*<.05), protein intake (−17 vs −6 g; *P*<.05), and carbohydrate intake (−107 vs −1 g; *P*<.05) of the participants in group DPI were statistically significant compared with the participants in group PI. There was a significant difference in the daily fat intake of the participants in group PI (−7 vs 3 kg; *P*<.05) compared with the participants in group C.

Compared with the food intake at time 1, the intake of cereals and potatoes (*P*<.001) and oil (*P*=.01) among the participants in group DPI at time 3 decreased. In group PI, the intake of vegetables (*P*=.02) and oil (*P*<.001) also decreased. Please refer to Table 3 for further details.

**Table 3:** Changes in dietary intake at time 1 and time 3 compared with baseline for the participants in all 3 groups (n=642).

			Group DPI^a^											Group PI^b^											Group C^c^											*F* test (*df*) or *z* score			*P* value
			Time 1		Time 3		MD^d^		*F* test (*df*) or *z* score		*P* value		Time 1			Time 3		MD		*F* test (*df*) or *z* score		*P* value		Time 1			Time 3		MD		*F* test (*df*) or chi-square (*df*)		*P* value						
Energy (kcal/d), mean (SD)^e^			2235 (636)		1654 (686)		−581 (799) ^f,g^		91.688 (236)		<.001^h^		2243 (827)			2118 (793)		−125 (993)		2.432 (202)		.12		2283 (832)			2200 (755)		−82 (1042)		1.095 (201)		.30		0.232			.79	
Protein (g/d), mean (SD)^e^			77 (29)		60 (40)		−17 (47)^f,g^		27.678 (236)		<.001^h^		77 (36)			71 (33)		−6 (42)		3.211 (202)		.07		76 (31)			74 (39)		−3 (47)		0.518 (201)		.47		0.006			.99	
Fat (g/d), mean (SD)^e^			75 (27)		67 (42)		−8 (45)^f^		6.256 (236)		.01^h^		80 (33)			73 (39)		−7 (46)^i^		3.923 (202)		.048^h^		77 (32)			80 (38)		3 (48)		0.906 (201)		.34		1.836			.16	
Carbohydrate (g/d), mean (SD)^e^			313 (104)		207 (103)		−107 (121)^f,g^		124.937 (236)		<.001^h^		302 (126)			301 (149)		−1 (172)		0.004 (202)		.95		318 (134)			314 (145)		−5 (182)		0.116 (201)		.73		1.024			.36	
**Food (g/d), median (IQR)^j^**																																							
	Cereals and potatoes	217 (143-305)		156 (106-234)		N/A^k^		−4.7 (236)		<.001^h^		290 (200-403)			257 (179-391)		N/A		−1.3 (202)		.19		313 (232-456)			300 (213-419)		N/A		−0.9 (201)		.33		61.098			<.001		
	Vegetables	405 (215-634)		354 (205-578)		N/A		−1.2 (236)		.24		357 (200-632)			292 (179-500)		N/A		−2.4 (202)		.02^h^		307 (178-535)			343 (216- 547)		N/A		−0.9 (201)		.36		7.724			.02^l^		
	Fruit	342 (202-501)		322 (190-543)		N/A		−0.1 (236)		.89		278 (170-476)			341 (195-495)		N/A		−1.7 (202)		.09		250 (124-496)			319 (178-472)		N/A		−1.9 (201)		.05		8.617			.01^l^		
	Livestock and poultry meat	80 (48-130)		71 (37-121)		N/A		−1.5 (236)		.14		71 (36-133)			73 (39-129)		N/A		−0.0 (202)		.98		74 (31-131)			85 (41-131)		N/A		−1.5 (201)		.15		2.090			.35		
	Aquatic product	35 (13-74)		43 (20-70)		N/A		−1.4 (236)		.16		35 (13-72)			41 (13-71)		N/A		−0.6 (202)		.56		30 (11-71)			43 (17-86)		N/A		−1.9 (201)		.05		0.523			.770		
	Dairy products	33 (0-200)		40 (0-200)		N/A		−0.5 (236)		.68		57 (0-200)			86 (0-200)		N/A		−0.6 (202)		.52		55 (0-200)			57 (0-200)		N/A		−0.6 (201)		.95		1.166			.56		
	Eggs	50 (35-55)		50 (43-52)		N/A		−0.9 (236)		.35		50 (38-57)			50 (48-53)		N/A		−0.2 (202)		.87		50 (29-54)			50 (40-54)		N/A		−0.8 (201)		.42		1.614			.45		
	Soybeans	32 (13-6)		29 (14-49)		N/A		−1.6 (236)		.12		36 (14-82)			33 (14-64)		N/A		−1.0 (202)		.31		29 (11-71)			35 (19-68)		N/A		−1.2 (201)		.25		2.424			.30		
	Oil	31 (19-43)		25 (15-38)		N/A		−2.8 (236)		.01^h^		35 (24-48)			29 (15-41)		N/A		−3.5 (202)		<.001^h^		32 (19-44)			31 (23-46)		N/A		−1.2 (201)		.22		7.555			.02^l^		

^a^Group DPI: remote dietary and physical activity intervention group.

^b^Group PI: remote physical activity intervention group.

^c^Group C: control group.

^d^MD: mean difference between time 1 and time 3.

^e^Values were compared using the method of repeated measurement analysis (ANOVA).

^f^Statistically significant differences existed in the mean difference between group DPI and group C.

^g^Statistically significant differences existed in the mean difference between group DPI and group PI.

^h^Statistically significant differences existed between time 1 and time 3 in the same group.

^i^Statistically significant differences existed in the mean difference between group PI and group C.

^j^Values were compared at time 1 and time 3 using Mann-Whitney *U* test and compared among the 3 groups using the Kruskal-Wallis test.

^k^N/A: not applicable.

^l^Statistically significant differences existed among the 3 groups at time 1.

### Changes in Physical Activity

Compared with physical activity at time 1, the physical activity related to transportation (group PI: 693 vs 597 MET-min/week, group C: 693 vs 594 MET-min/week; all *P*<.05) and physical activity related to housework and gardening (group PI: 11 vs 0 MET-min/week, group C: 11 vs 4 MET-min/week; all *P*<.05) were enhanced at time 3. At time 3, there was no statistical difference in the distribution of the physical activity levels of the participants in each group (all *P*>.05). Please refer to Table 4 for further details.

**Table 4:** Changes in physical activity at time 1 and time 3 compared with baseline for the participants in all 3 groups.

		Group DPI^a^							Group PI^b^								Group C^c^								*z* score or hi-square (*df*)			*P* value
		Time 1 (n=237)	Time 3 (n=237)	*z* score or chi-square (*df*)		*P* value		Time 1 (n=203)		Time 3 (n=203)	*z* score or chi-square (*df*)		*P* value		Time 1 (n=202)			Time 3 (n=202)	*z* score or hi-square (*df*)		*P* value							
**Physical activity (MET^d^-min/week)**																												
	Total, median (IQR)^e^	2391 (1003-4253)	2689 (1248-4194)	−0.959		.34		2044 (677-3850)		2418 (858-5011)	−1.614		.11		1644 (713-3365)			2079 (896-4020)	−1.835		.07		8.085			.02^f^		
	Occupation related, median (IQR)^e^	0 (0-0)	0 (0-9)	−0.364		.72		0 (0-0)		0 (0-0)	−0.718		.47		0 (0-0)			0 (0-0)	−1.064		.29		1.030			.60		
	Transportation, median (IQR)^e^	693 (0-1386)	693 (297-1386)	−1.722		.09		597 (0-1386)		693 (157-1672)	−2.496		.01^g^		594 (0-1386)			693 (198-1411)	−2.202		.03^g^		0.936			.63		
	Housework and gardening, median (IQR)^e^	11 (0-25)	11 (0-25)	−0.409		.68		0 (0-25)		11 (0-25)	−2.967		.003^g^		4 (0-25)			11 (0-25)	−2.376		.02^g^		8.732			.01^f^		
	Leisure related, median (IQR)^e^	544 (0-1706)	594 (0-1677)	−0.266		.79		396 (0-1467)		396 (0-1901)	−0.751		.45		297 (0-1285)			570 (0-1386)	−1.649		.099		5.200			.07		
**Physical activity level, n (%)^h^**					3.7 (236)		.15					2.3 (202)		.32						4.1 (201)		.13		3.9 (641)			.42	
	Low	50 (20.2)	32 (13.6)					59 (24.4)		44 (21.9)					61 (25)			36 (18.2)										
	Middle	95 (38.3)	98 (41.5)					93 (38.4)		68 (33.8)					101 (41.4)			80 (40.4)										
	High	103 (41.5)	106 (44.9)					90 (37.2)		89 (44.3)					82 (33.6)			82 (41.4)										

^a^Group DPI: remote dietary and physical activity intervention group.

^b^Group PI: remote physical activity intervention group.

^c^Group C: control group.

^d^MET: metabolic equivalent.

^e^Values were compared at time 1 and time 3 using Mann-Whitney *U* test and compared among 3 groups using Kruskal-Wallis test.

^f^Statistically significant differences existed among the 3 groups at time 1.

^g^Statistically significant differences existed between time 1 and time 3 in the same group.

^h^Values were compared using chi-square test.

### Changes in the Indexes Related to Weight Control

Compared with the indexes related to weight control at time 1, the body weight, BMI, and waist circumference of group DPI were significantly decreased (all *P*<.05). Comparing the mean differences between time 2 and time 1 (MD_12_) in the indexes related to weight control among the 3 groups, there were statistically significant MD_12_ in body weight (−1.56 vs −0.86 vs −0.66 kg; *P*<.001) and BMI (−0.61 vs −0.33 vs −0.27 kg/m^2^; *P*<.001). Comparing the mean differences between time 3 and time 1 (MD_13_) in the indexes related to weight control among the 3 groups, there were statistically significant MD_13_ in body weight (−4.11 vs −1.01 vs −0.83 kg; *P*<.001), BMI (−1.61 vs −0.40 vs −0.33 kg/m^2^; *P*<.001), and waistline (−2.76 vs −0.12 vs 0.47 cm; *P*<.001; Table 5).

**Table 5:** Changes in the indexes related to weight control at time 1, time 2, and time 3 compared with baseline for the participants in all 3 groups^a^.

		Group DPI^b^	Group PI^c^	Group C^d^	*F* test (*df*)	*P* value
**Weight (kg)**						
	Time 1, mean (SD)	70.45 (9.12)^e^	70.56 (9.68)	70.02 (10.19)^f^	0.006 (641)	.994
	Time 2, mean (SD)	68.90 (8.91)	69.70 (9.45)	69.85 (10.27)	0.643 (641)	.53
	Time 3, mean (SD)	66.35 (8.85)^g^	69.55 (9.56)^h^	69.68 (10.08)	8.881 (641)	<.001^i^
	*F* test (*df*)	12.682 (236)	0.655 (202)	0.377 (201)	N/A^j^	N/A
	*P* value	<.001^i^	.52	.69	N/A	N/A
	MD_12_^k^ (time 2 − time 1)	−1.56 (1.22)^f,h^	−0.86 (1.30)	−0.66 (3.53)	9.829 (641)	<.001^i^
	MD_13_^l^ (time 3 − time 1)	−4.11 (1.45)^f,h^	−1.01 (1.70)	−0.83 (2.00)	257.541 (641)	<.001^i^
**BMI (kg/m^2^)**						
	Time 1, mean (SD)	27.61 (2.23)^e^	27.66 (2.99)	27.83 (2.75)^f^	0.382 (641)	.68
	Time 2, mean (SD)	27.01 (2.19)^m,h^	27.33 (2.92)	27.56 (2.75)	2.506 (641)	.08
	Time 3, mean (SD)	26.00 (2.22)^g,f,n^	27.26 (2.90)^h^	27.49 (2.66)	21.432 (641)	<.001^i^
	*F* test (*df*)	32.139 (236)	1.075 (202)	0.852 (201)	N/A	N/A
	*P* value	<.001^i^	.34	.43	N/A	N/A
	MD_12_ (time 2 − time 1)	−0.61 (0.47)^f,h^	−0.33 (0.51)	−0.27 (1.35)	9.981 (641)	<.001^i^
	MD_13_ (time 3 − time 1)	−1.61 (0.57)^f,h^	−0.40 (0.67)	−0.33 (0.78)	253.683 (641)	<.001^i^
**Waistline (cm)**						
	Time 1, mean (SD)	93.82 (8.24)^e^	95.05 (8.14)	95.31 (7.91)^f^	2.158 (641)	1.00
	Time 2, mean (SD)	93.38 (7.91)	94.73 (8.10)	95.02 (7.76)	2.763 (641)	.06
	Time 3, mean (SD)	91.06 (8.55)^g^	94.91 (8.10)^h^	95.37 (7.86)	21.043 (641)	<.001^i^
	*F* test (*df*)	7.698 (236)	0.080 (202)	0.487 (201)	N/A	N/A
	*P* value	<.001^i^	.92	.62	N/A	N/A
	MD_12_ (time 2 − time 1)	−0.44 (5.17)	−0.32 (4.67)	−0.30 (5.07)	0.057 (641)	.94
	MD_13_ (time 3 − time 1)	−2.76 (5.60)^f,h^	−0.12 (4.84)	0.47 (5.16)	24.219 (641)	<.001^i^
**Waist-hip ratio^m^**						
	Time 1, mean (SD)	0.92 (0.06)	0.93 (0.06)	0.93 (0.05)^e,f^	1.440 (641)	.24
	Time 2, mean (SD)	0.94 (0.07)	0.94 (0.07)	0.94 (0.06)	0.840 (641)	<.001^i^
	Time 3, mean (SD)	0.93 (0.07)	0.94 (0.06)	0.94 (0.06)	3.583 (641)	.03^i^
	*F* test *(df)*	2.980 (236)	2.613 (202)	4.432 (201)	N/A	N/A
	*P* value	.05	.07	.01^i^	N/A	N/A
	MD_12_ (time 2 − time 1)	0.01 (0.06)	0.01 (0.06)	0.01 (0.06)	0.589 (641)	.56
	MD_13_ (time 3 − time 1)	0.01 (0.06)	0.01 (0.06)	0.02 (0.06)	1.261 (641)	.28

^a^Values presented as the mean (SD) were compared using the method of repeated measurement analysis (ANOVA).

^b^Group DPI: remote dietary and physical activity intervention group.

^c^Group PI: remote physical activity intervention group.

^d^Group C: control group.

^e^Statistically significant differences existed between time 1 and time 2.

^f^Statistically significant differences existed in the mean difference between group DPI and group C.

^g^Statistically significant differences existed between time 2 and time 3.

^h^Statistically significant differences existed in the mean difference between group DPI and group PI.

^i^Statistically significant differences: *P*<.05.

^j^N/A: not applicable.

^k^MD_12_: mean difference between time 1 and time 2.

^l^MD_13_: mean difference between time 1 and time 3.

^m^Statistically significant differences existed between time 1 and time 3.

^n^Statistically significant differences existed in the mean difference between group PI and group C.

### Changes in the Indexes Related to Health Benefits

Compared with the indexes related to health benefits at time 1, the SBP, DBP, TC, TG, FBG, and HbA_1c_ of group DPI significantly decreased (all *P*<.05). Compared with the indexes related to health benefits at time 1, the SBP of groups DPI and C significantly decreased (all *P*<.05), and the HDL-C of group C significantly decreased (*P*=.04). Comparing the mean differences between time 2 and time 1 (MD_12_) in the indexes related to body health benefits among the 3 groups, there were statistically significant MD_12_ in TG (−0.06 vs −0.12 vs 0.32 mmol/L; *P*=.003) and LDL-C (−0.02 vs −0.11 vs −0.13 mmol/L; *P*=.03). Comparing the mean differences between time 3 and time 1 (MD_13_) in the indexes related to body health benefits among the 3 groups, there were statistically significant MD_13_ in SBP (−8.15 vs −3.04 vs −3.80 mm Hg; *P*=.001), TG (−0.48 vs 0.11 vs 0.18 mmol/L; *P*<.001), HDL-C (−0.00 vs −0.04 vs −0.07 mmol/L; *P*<.001), fasting plasma glucose (−0.77 vs −0.43 vs −0.14 mmol/L; *P*<.001), and HbA_1c_ (−0.19 vs −0.08 vs −0.07 mmol/L; *P*=.02; Table 6).

**Table 6:** Changes in the indexes related to health benefits at time 1, time 2, and time 3 compared with baseline for the participants in all 3 groups^a^.

			Group DPI^b^		Group PI^c^		Group C^d^		*F* test (*df*)		*P* value
**SBP^e^ (mmHg)**											
	Time 1, mean (SD)	133.73 (16.42)^f^		131.22 (16.92)^f^		132.15 (19.65)^f^		1.147 (641)		.32	
	Time 2, mean (SD)	129.90 (17.55)^g^		126.65 (16.16)		127.74 (16.68)		2.138 (641)		.12	
	Time 3, mean (SD)	125.58 (16.33)^h^		128.18 (16.39)		128.35 (16.90)^g^		1.969 (641)		.14	
	*F* test (*df*)	13.994 (236)		4.041 (202)		3.647 (201)		N/A^i^		N/A	
	*P* value	<.001^j^		.02^j^		.03^j^		N/A		N/A	
	MD_12_^k^ (time 2 − time 1)	−3.84 (13.10)		−4.57 (14.39)		−4.41 (15.33)		0.165 (641)		.85	
	MD_13_^l^ (time 3 − time 1)	−8.15 (14.33) ^f,h^		−3.04 (15.69)^m^		−3.80 (15.52)^n^		7.404 (641)		.001^j^	
**DBP ^o^ (mmHg)**											
	Time 1	74.16 (10.92)		75.11 (11.11)		73.93 (11.32)		0.654 (641)		.52	
	Time 2	72.97 (10.832)		73.48 (10.78)		72.21 (10.80)		0.716 (641)		.49	
	Time 3	71.76 (10.02)^h^		74.13 (10.59)^m^		71.92 (10.47)^p^		3.431 (641)		.03^j^	
	*F* test (*df*)	3.041 (236)		1.167 (202)		2.032 (201)		N/A		N/A	
	*P* value	.048^j^		.31		.13		N/A		N/A	
	MD_12_ (time 2 − time 1)	−1.18 (6.88)		−1.63 (8.67)		−1.73 (8.46)		0.289 (641)		.75	
	MD_13_ (time 3 − time 1)	−2.40 (8.00)		−0.99 (9.85)		−2.02 (7.88)		1.545 (641)		.21	
**TC^q^ (mmol/L)**											
	Time 1, mean (SD)	4.92 (0.99)		5.04 (1.00)		4.96 (1.02)		0.759 (641)		.47	
	Time 2, mean (SD)	4.85 (0.96)^g^		4.92 (0.98)		4.89 (0.99)		0.239 (641)		.79	
	Time 3, mean (SD)	4.67 (0.95)^h^		4.89 (0.99)^m^		4.81 (1.00)		3.056 (641)		.048^j^	
	*F* test (*df*)	4.403 (236)		1.211 (202)		1.232 (201)		N/A		N/A	
	*P* values	.01^j^		.30		.29		N/A		N/A	
	MD_12_ (time 2 − time 1)	−0.07 (0.57)		−0.12 (0.61)		−0.07 (0.67)		0.503 (641)		.61	
	MD_13_ (time 3 − time 1)	−0.25 (0.75)		−0.14 (0.72)		−0.16 (0.76)		1.467 (641)		.23	
**TG^r^ (mmol/L)**											
	Time 1, mean (SD)	1.82 (1.01)		1.99 (1.34)		1.86 (1.13)		1.183 (641)		.31	
	Time 2, mean (SD)	1.76 (0.99)^g^		2.10 (1.52)		2.19 (1.91)^n,p^		5.118 (641)		.01^j^	
	Time 3, mean (SD)	1.34 (0.62)^h^		2.10 (1.92)^m^		2.04 (1.37)^n^		21.172 (641)		<.001^j^	
	*F* test (*df*)	20.58 (236)		0.342 (202)		2.359 (201)		N/A		N/A	
	*P* value	<.001^j^		.71		.10		N/A		N/A	
	MD_12_ (time 2 − time 1)	−0.06 (0.85)^f^		−0.12 (0.77)		0.32 (1.71)^n^		5.873 (641)		.003^j^	
	MD_13_ (time 3 − time 1)	−0.48 (0.78) ^f,h^		0.11 (1.08)^m^		0.18 (0.98)^n^		32.964 (641)		<.001^j^	
**HDL-C^s^ (mmol/L)**											
	Time 1	1.30 (0.32)		1.27 (0.30)		1.27 (0.30)		0.549 (641)		.58	
	Time 2	1.27 (0.31)		1.24 (0.30)		1.22 (0.28)		1.532 (641)		.22	
	Time 3	1.29 (0.29)		1.24 (0.30)^b^		1.23 (0.28)^h,n^		6.41 (641)		.002^j^	
	*F* test (*df*)			0.919 (202)		3.332 (201)		N/A		N/A	
	*P* value	.60		.40		.04^j^		N/A		N/A	
	MD_12_ (time 2 − time 1)	−0.03 (0.15)		−0.03 (0.15)		−0.05 (0.16)		0.976 (641)		.38	
	MD_13_ (time 3 − time 1)	−0.00 (0.17)^e^		−0.04 (0.16)^m^		−0.07 (0.16)^n,p^		9.542 (641)		<.001^j^	
**LDL-C^t^ (mmol/L)**											
	Time 1, mean (SD)	2.30 (0.87)		3.08 (0.93)		3.08 (0.99)		0.579 (641)		.56	
	Time 2, mean (SD)	3.02 (0.89)		2.98 (0.92)		2.95 (0.93)		0.283 (641)		.75	
	Time 3, mean (SD)	2.97 (0.88)		2.96 (0.93)		2.92 (0.96)		0.173 (641)		.84	
	*F* test (*df*)	0.152 (236)		1.089 (202)		1.486 (201)		N/A		N/A	
	*P* value	.86		.34		.23		N/A		N/A	
	MD_12_ (time 2 − time 1)	0.02 (0.60)^f^		−0.11 (0.63)^m^		−0.13 (0.62)^n,p^		3.521 (641)		.03^J^	
	MD_13_ (time 3 − time 1)	−0.03 (0.75)		−0.13 (0.72)		−0.15 (0.75)		1.847 (641)		.16	
**FPG^u^ (mmol/L)**											
	Time 1, mean (SD)	6.97 (2.15)		6.89 (2.23)		6.85 (2.17)		0.169 (641)		.85	
	Time 2, mean (SD)	6.70 (2.09)^g^		6.75 (2.57)		6.86 (2.22)		0.280 (641)		.76	
	Time 3, mean (SD)	6.20 (1.47)^h^		6.46 (1.98)		6.70 (2.08)^n^		4.096 (641)		.02^j^	
	*F* test (*df*)	9.623 (236)		1.886 (202)		0.324 (201)		N/A		N/A	
	*P* value	<.001^j^		.15		.72		N/A		N/A	
	MD_12_ (time 2 – time 1)	−0.27 (1.46)		−0.14 (1.50)		0.01 (1.19)		2.232 (641)		.11	
	MD_13_ (time 3 – time 1)	−0.77 (1.18) ^f,h^		−0.43 (1.20)		−0.14 (1.56)^n^		12.364 (641)		<.001^j^	
**HbA_1c_^v^ (%)**											
	Time 1, mean (SD)	6.37 (0.98)		6.35 (1.19)		6.44 (1.15)		0.418 (641)		.66	
	Time 2, mean (SD)	6.37 (0.97)^g^		6.37 (1.40)		6.46 (1.12)		0.406 (641)		.67	
	Time 3, mean (SD)	6.18 (0.83)^h^		6.27 (1.02)		6.38 (1.06)		2.269 (641)		.10	
	*F* test (*df*)	3.22 (236)		0.397 (202)		0.305 (201)		N/A		N/A	
	*P* value	.04^j^		.67		.74		N/A		N/A	
	MD_12_ (time 2 − time 1)	−0.00 (0.31)		0.03 (0.70)		0.02 (0.35)		0.178 (641)		.84	
	MD_13_ (time 3 − time 1)	−0.19 (0.39)^f^		−0.08 (0.42)^m^		−0.07 (0.66)^n^		4.085 (641)		.02^j^	

^a^Values presented as the mean (SD) were compared using the method of repeated measurement analysis (ANOVA).

^b^Group DPI: remote dietary and physical activity intervention group.

^c^Group PI: remote physical activity intervention group.

^d^Group C: control group.

^e^SBP: systolic blood pressure.

^f^Statistically significant differences existed between time 1 and time 2.

^g^Statistically significant differences existed between time 2 and time 3.

^h^Statistically significant differences existed between time 1 and time 3.

^i^N/A: not applicable.

^j^Statistically significant differences: *P*<.05.

^k^MD_12_: mean difference between time 1 and time 2.

^l^MD_13_: mean difference between time 1 and time 3.

^m^Statistically significant differences existed in the mean difference between the group DPI and group PI.

^n^Statistically significant differences existed in the MD between group DPI and group C.

^o^DSP: diastolic blood pressure.

^p^Statistically significant differences existed in the MD between group PI and group C.

^q^TC: total cholesterol.

^r^TG: triglyceride.

^s^HDL-C: high-density lipoprotein cholesterol

^t^LDL-C: low-density lipoprotein cholesterol.

^u^FPG: fasting plasma glucose.

^v^HbA_1c_: hemoglobin A_1c_.

## Discussion

### Principal Findings

In this study, remote dietary and physical activity interventions improved dietary intake among participants with overweight or obesity, were beneficial for weight control, and had potential health benefits. The remote management system mode that included “health information collection—health assessment—guidance and feedback—follow-up” was feasible among older people and effective in this study.

The key to weight control is balancing dietary intake and energy expenditure. Although focusing on weight changes, this study also analyzed the impact of a remote management mode on the behavioral changes in dietary intake and physical activity. The results indicated that after 3 months of a remote intervention, the daily total energy intake and the intake of 3 macronutrients were significantly reduced in group DPI compared with group C, and the daily fat intake was reduced in group PI compared with group C. When comparing the intake of various foods, it was found that the intake of cereals, potatoes, and oils in group DPI was significantly reduced. Compared with group C, the intake of cereal and potato foods in group PI was also significantly reduced. The remote management system based on the mode of “health information collection—health assessment—guidance and feedback—follow-up” had a relatively mature monitoring and interaction mechanism, and it provided support and encouragement to the participants to promote changes in dietary intake behavior [29]. There was no significant difference in the physical activity of each group before and after the intervention. The reason may be that the participants were older adults, and it was not mandatory for them to complete all physical activities. In addition, the intervention period was relatively short, which may be an important reason for the lack of significant improvement in the physical activity of the participants. A systematic review of 27 studies also found that stand-alone interventions using an app only, or multicomponent interventions, including an app as one of several intervention components, helped improve the dietary pattern, increase physical activity, and reduce the sedentary behavior of the population [30]. Another systematic review and meta-analysis included 28 studies (N=7454) and showed that physical activity interventions involving smartphone apps or activity trackers with the function of automated and continuous self-monitoring and feedback were effective in promoting physical activity [19]. A similar conclusion was reached in a systematic review and meta-analysis that included 9 studies (N=1740), which demonstrated the effectiveness of smartphone apps as the primary or sole component of a physical activity intervention [31].

This study also explored the effects of remote dietary and physical activity interventions on weight management. The results indicated that, at the end of the intervention, compared with groups PI and C, the weight-related indicators of the participants in group DPI, including body weight, BMI, and waist circumference, were significantly decreased. In recent years, an increasing number of studies have evaluated the impact and effect of remote mobile technology combined with wearable devices on weight control. A systematic review and meta-analysis including 20 studies indicated that behavior-related weight management interventions using mobile health (mHealth) lifestyle self-monitoring were effective and had relatively higher adherence in the short term [32]. A 12-week, assessor-blind, parallel-group, waitlist-controlled, and randomized trial demonstrated that receiving a weight loss program with dietary coaching by a certified nutrition professional via group chat delivered on a smartphone app was effective for weight loss, and participants were actively involved [33]. An 8-week intervention program using a mobile platform recruited 150 adults with overweight or obesity, and the results suggested that the combination of an mHealth care app consisting of food, exercise, and weight logging; in-app group activities; in-app articles; messages from the coaches; and offline care by health care providers was effective for weight management with a resulting decrease in weight, BMI, and waistline [34]. A pilot randomized controlled trial compared the effects of a paper diary and the smartphone app “My Meal Mate” incorporating goal setting, self-monitoring of dietary intake and activity, and feedback on weight loss among 128 overweight volunteers and indicated that the participants in the smartphone app intervention group had fewer missing data, higher adherence rate, higher weight loss, and a greater BMI decrease [35]. A systematic review of 25 studies showed that for middle-aged and older people with BMI≥25 kg/m^2^, a short-term (6-month) weight management plan combined with wearable devices, such as activity trackers, may be better than traditional weight management [20]. This study showed no statistical difference between groups PI and C. Therefore, whether the weight control effect of group DPI was completely attributable to the remote dietary intervention or whether there was an interaction between the remote dietary and remote physical activity interventions requires further research.

In this study, remote dietary and physical activity interventions demonstrated potential health benefits, including lower blood pressure, TG, FBG, HDL-C, and HbA_1c_. A 3-month randomized controlled study explored the possible effects of internet-based interventions among 105 adults with overweight or obesity who had hypertension and found that a self-administered, internet-based exercise and nutritional education intervention significantly reduced BMI, body fat mass, and blood glucose [36]. In a randomized controlled study of 244 patients with diabetes who had uncontrolled systolic hypertension over a year, it was suggested that the home blood pressure telemonitoring system with self-care support on the smartphone effectively improved blood pressure [21]. A randomized controlled study with 349 patients with hypertension showed that a remote “Internet+” interactive management mode with blood pressure intervention was effective for the improvement of blood pressure [23]. A randomized controlled trial demonstrated that compared with the standard care group, TC and LDL-C levels were decreased in the intervention group using the smartphone app Perx, which included customized reminders and gamified interactions to reward verified medication adherence [37]. In a 4- to 6-week randomized controlled trial of 267 patients with diabetes, heart disease, or both, it was concluded that an intervention of telemonitoring and smartphone-based health coaching supported by a remote monitoring system could reduce the waistline significantly, but blood pressure reduction was not statistically significant [38]. A meta-analysis of 9 studies demonstrated that the use of apps permitting the structured monitoring of health parameters and feedback effectively improved lifestyle and decreased HbA_1c_ [39]. A study conducted among 12,530 patients with type 2 diabetes found that an mHealth app with health education, rehabilitation training, medication reminders, and dietary guidance controlled glycemic levels [40]. A randomized controlled study conducted among 120 older individuals with diabetes demonstrated that the continuous application of a mobile app including health education effectively improved blood glucose levels, enhanced self-management ability, and reduced complications in the participants [41]. The results of this study also preliminarily confirmed that the remote management system and related remote interventions were feasible for the older population.

This study has some strengths and weaknesses. With the help of intelligent equipment such as smartphones, remote health management can efficiently realize the remote monitoring of older users without increasing labor costs and without time and geographical constraints. These advantages cannot be ignored, but there are also some difficulties and problems. The implementation of remote management involves the use of relevant apps and intelligent monitoring equipment, which may be a problem and cause difficulty for the older population. For them, smart devices should be visually clear and personalized with simple navigation, be easily operatable, and monitor the applicability and motivation during remote health management procedures. The interface of the smartphone app used in this study was clear and convenient for the older people to operate. In addition, many methods to encourage behavior change were established in the intervention process, including web-based education, daily weight monitoring, weekly monitoring of blood pressure and blood glucose, setting goals, motivation, and timely feedback. In this study, WeChat groups were established, which helped the participants obtain social and emotional support from each other, thus improving their enthusiasm and follow-up. Since the emergence of remote medical technology, many scholars have studied the effects and prospects of remote health intervention or management as applied to individual health management, including that of children, adolescents, and adults; however, few studies have focused on the older population. This study focused on older people with overweight and obesity, and no similar study was found in China. Regarding the weaknesses, the duration of the study was only 3 months, and the sustainability of the effect after the intervention was not evaluated. There was no traditional management group in the study, so it was impossible to compare the effects between the remote management mode and traditional management mode.

The results of this study can be generalized, and the remote health management mode and intervention measures adopted have relatively good practicability. Therefore, more and better-designed studies are needed to verify the effectiveness of this intervention and the sustainability of related effects and to provide ideas and references for the remote management of chronic diseases in the older population. The future development direction will be to combine remote and intelligent health care with traditional offline health management to explore the best universal health management scheme for conveniently and economically promoting the health of the older people.

### Conclusions

The remote smartphone-based dietary and physical activity interventions helped older people with overweight and obesity reduce their intake of total dietary energy but did not significantly improve their physical activity. They had a better effect on weight control and improved blood pressure, blood glucose, and blood lipids. This study preliminarily confirmed the feasibility and effectiveness of the remote management mode of “health information collection—health assessment—guidance and feedback—follow-up” in the older population. It is recommended that this remote smartphone-based dietary and physical activity intervention pattern be further applied to promote health management in the older population.

## Appendix

Multimedia Appendix 1 CONSORT eHEALTH Checklist (V 1.6.1).

## Abbreviations

DBP: diastolic blood pressure
FBG: fasting blood glucose
Group C: control group
Group DPI: remote dietary and physical activity intervention group
Group PI: remote physical activity intervention group
HbA_1c_: hemoglobin A_1c_
HDL-C: high-density lipoprotein cholesterol
LDL-C: low-density lipoprotein cholesterol
MD12: mean difference between time 1 and time 2
MD13: mean difference between time 1 and time 3
MET: metabolic equivalent
mHealth: mobile health
SBP: systolic blood pressure
TC: total cholesterol
TG: triglyceride
Time 1: baseline
Time 2: day 45
Time 3: day 90

## Data Availability

The corresponding author will provide the data used in the manuscript, code book, and analytic code in a deidentified form, which are available to editors upon request, either before or after publication.
